# SYSTEMATIC REVIEW OF REHABILITATION IN SKILLED NURSING FACILITIES FOR PERSONS LIVING WITH DEMENTIA

**DOI:** 10.1093/geroni/igad104.3614

**Published:** 2023-12-21

**Authors:** Erica Frechman, Charles Semelka, Valerie Freeman

**Affiliations:** Atrium Health Wake Forest Baptist, Winston-Salem, North Carolina, United States; Wake Forest University School of Medicine, Winston Salem, North Carolina, United States; Atrium Health, Charlotte, North Carolina, United States

## Abstract

We conducted a systematic review of published literature though April 2023 to identify the experiences and outcomes of persons living with dementia (PLWD) undergoing post-acute care rehabilitation in skilled nursing facilities (SNF). A systematic search for studies published in English was carried out in eight databases including PubMed, Scopus, Google Scholar, Embase, PsycINFO, CINAHL, Cochrane Library, and Web of Science. Two reviewers independently screened articles and conducted a quality appraisal of selected studies. Forty-one articles met inclusion criteria. There was heterogeneity of study designs with most articles (n=33) being observational and cross-sectional, while randomized clinical trials (n=3) and qualitative studies (n=5) were less represented. Certain themes emerged from the results regarding patient outcomes and experiences. Measured outcomes demonstrated increased health service utilization, varied functional outcomes, and suboptimal patient care. Patient experiences included significant challenges with health care transitions, gaps in healthcare worker and caregiver knowledge & education, emphasis on alternate models of care, and importance of goal alignment. Overall, this systematic review provides a comprehensive understanding of the outcomes and experiences of post-acute care rehabilitation in SNFs for PLWD. The findings highlight the need for alternative care pathways, improved healthcare transitions, tailored rehabilitation approaches, enhanced dementia training for healthcare professionals, and person-centered goal setting. By addressing these critical aspects, future interventions can be developed to improve the delivery of post-acute care for PLWD and enhance their health outcomes.

